# New Drug Developments for Neuromuscular Blockade and Reversal: Gantacurium, CW002, CW011, and Calabadion

**DOI:** 10.1007/s40140-018-0262-9

**Published:** 2018-03-22

**Authors:** Hans Donald de Boer, Ricardo Vieira Carlos

**Affiliations:** 1 Department of Anesthesiology and Pain Medicine, Martini General Hospital Groningen, PO Box 30033, 9700 RM Groningen, the Netherlands; 2 Department of Anesthesiology, Child Institute, Hospital das Clinicas, Sao Paulo University Medical School, Sao Paulo, Brazil

**Keywords:** Gantacurium, CW002, CW011, Calabadion

## Abstract

**Purpose of Review:**

The purpose of this chapter is to provide a brief review of the literature on the recent developments in neuromuscular blockade and reversal agents.

**Recent Findings:**

Novel drug development resulted in pharmacological advancements in neuromuscular management and led to a new series of compounds, chlorofumarates, such as gantacurium, CW002, and CW011. These drugs have a fast onset and rapid to intermediate duration of action and can be rapidly reversed by l-cysteine adduction without side effects that are commonly observed with anticholinesterase reversal drugs. Another new advancement is the development of a new class of reversal drugs, the calabadions. These drugs are able to reverse both steroidal and non-steroidal non-depolarizing neuromuscular blocking drugs rapidly.

**Summary:**

Recent advancements in neuromuscular blocking agents and reversal drugs have shown promise in improving safety of management of neuromuscular blockade. Preclinical and clinical studies are discussed. However, to date these new drugs are not yet available for clinical use.

## Introduction

Over 75 years ago, Harold Griffith and Enid Johnson published their famous paper on the use of curare in general anesthesia [1]. The introduction of curare allowed adequate muscle relaxation at a lighter, and therefore better-tolerated, depth of general anesthesia. During these last 75 years, several new compounds were developed to maintain neuromuscular block. One important area of research was the development of a short-acting non-depolarizing neuromuscular blocking drug with a fast onset of effect, short duration of action, which was independent of end-organ metabolism and allowed for rapid and complete reversal. Actually, this research reflected a continuing search for a replacement for succinylcholine. For this reason, an ideal neuromuscular blocking drug was defined: the profile of an ideal neuromuscular blocking drug should meet the criteria developed by Savarese and Kitz and should distinguish among others three types of drugs: short, intermediate, and long-acting [2]. However, to date, a short-acting non-depolarizing neuromuscular blocking drug with a fast onset, comparable pharmacodynamically to succinylcholine, but without the well-known undesirable side effects, is still not available for clinical use. Furthermore, in 1954, Beecher and Todd reported a study of the mortality associated with anesthesia and surgery, in which they found that mortality rates were six times higher when neuromuscular blocking drugs were used and that 63% of the deaths were caused by respiratory failure [3]. Since then, many publications have reported the risks of using neuromuscular blocking drug and the concomitant high incidence of residual neuromuscular block [4, 5••]. Therefore, reversal of neuromuscular block is essential when using these drugs.

Novel drug development resulted in pharmacological advancements in neuromuscular management and led to a new series of compounds, chlorofumarates as new neuromuscular blocking drugs and calabadion and l-cysteine adduction for reversal of neuromuscular block [6, 7].

This review will highlight the recent advancements in the field of neuromuscular blocking drugs and reversal of neuromuscular blockade.

## Recent Advancements of Neuromuscular Blocking Drugs

### Gantacurium

Gantacurium was the first compound of this new class of neuromuscular blocking drugs, the asymmetric mixed-onium chlorofumarates. Gantacurium is an asymmetric enantiomeric isoquinolinium diester of chlorofumaric acid [6, 7]. Gantacurium, like cisatracurium, is a single isomer as atracurium and mivacurium are consisting of a mixture of isomers [8]. Gantacurium is an ultra-short acting non-depolarizing neuromuscular blocking drug with a rapid onset and a wide safety margin [8–10]. As cisatracurium and atracurium are inactivated by Hofmann elimination, gantacurium is metabolized by chemical degradation which involves cysteine adduction (fast process) and pH-sensitive hydrolysis (slow process) [8, 9]. Cysteine adduction results in replacement of chlorine by cysteine whereafter a heterocyclic ring is formed which cannot longer interact with the postjunctional acetylcholine receptor. Therefore, the metabolites of gantacurium showed no neuromuscular properties [8, 9]. Moreover, there is no renal and hepatic involvement in the elimination of gantacurium [7–9]. Reversal of a gantacurium-induced neuromuscular block is possible with administration of cysteine [11]. This first drug in class showed promising results in animal and human studies which will be discussed.

#### Preclinical Studies

Preclinical studies in Rhesus monkeys, in which gantacurium was compared with mivacurium, showed that the potencies between gantacurium and mivacurium were identical [8]. The 3× ED_95_ of gantacurium and mivacurium was in both drugs 0.16 mg/kg. The onset was significantly faster compared with mivacurium. Administration of 0.18 mg/kg gantacurium resulted in a spontaneous recovery of a train-of-four ration ≥ 0.9 within 10 min. The duration at doses of 3× ED_95_ of either gantacurium or mivacurium to 95% twitch recovery was 8.5 ± 0.5 vs 22 ± 2.6 min respectively. Further increasing of the dose of gantacurium (up to 3.2 mg/kg) showed no changes in recovery intervals [8]. Doses higher than 6.4 mg/kg resulted in a longer duration (1.5–3.0 min), but no cumulative effects were observed. The spontaneous recovery from a neuromuscular block induced by gantacurium was shorter than that for a mivacurium-induced neuromuscular block. These results were confirmed in cats [8, 10, 12].

Interestingly, while gantacurium is shorter acting than succinylcholine in dogs, the results of studies in other animal species (Rhesus monkeys and cats) with gantacurium showed comparable neuromuscular properties. This raises the question whether gantacurium might replace succinylcholine in the future [8, 12].

In preclinical studies, gantacurium in a dose of 25–50× ED_95_ showed a 10–25% decrease in arterial pressure and minimal histamine release [8, 10, 11]. This was confirmed in beagles. No pulmonary vasoconstriction or bronchoconstriction was observed in these animals. Based on the promising preclinical studies, gantacurium was investigated in humans.

#### Human Studies

In human volunteers, the ED_95_ of gantacurium was 0.19 mg/kg [9]. The onset of 1× ED_95_ gantacurium was less than 3 min and could be shortened to approximately 1.5 min by increasing the dose to 4× ED_95_. At these doses, the duration of action of gantacurium (recovery to train-of-four of ≥ 0.90) was approximately 15 min. In this study, transient cardiovascular side effects were observed at doses of 3× ED_95_ or higher. Furthermore, humans showed significant histamine release when gantacurium was administered in doses of 4× ED_95_ [9, 13, 14]. However, at lower doses (2.5× ED_95_), there was no evidence of histamine release [9, 13, 14]. The margin of safety for histamine release, therefore, appears improved over that of mivacurium. However, there have been attempts to change the chemical structure of gantacurium in order to stabilize membranes of the molecule to alter histamine release, but to date, these attempts were not successful [14].

These results confirmed the promising characteristics of gantacurium in humans. The neuromuscular properties of gantacurium were comparable with the depolarizing neuromuscular blocking drug succinylcholine. Gantacurium in doses of 2–3× ED_95_ resulted in a 100% neuromuscular block at the laryngeal adductors and larynx in 1 min, whereas succinylcholine reached its maximal effect in 0.8 min [9, 13]. The spontaneous recovery of a neuromuscular block induced by gantacurium 2× ED_95_ was almost identical compared with the spontaneous recovery of a succinylcholine-induced neuromuscular block [13]. Therefore, based on these results, gantacurium seems to have nearly identical neuromuscular properties of succinylcholine without the unwanted side effects of this depolarizing neuromuscular blocking drug [9, 15••]. Further research is needed to confirm this preliminary conclusion. However, despite the results in preclinical and clinical studies, gantacurium is not available in clinical practice.

#### Reversal of Gantacurium-Induced Neuromuscular Blockade

Spontaneous recovery of a gantacurium-induced neuromuscular block is rapid. However, as gantacurium is a non-depolarizing neuromuscular blocking drug, gantacurium can be reversed with cholinesterase inhibitors [12]. As the spontaneous recovery of gantacurium is rapid, the most suitable drug for reversal is edrophonium as the peak effect of this reversal drug is less than 2 min. In humans, edrophonium was able to decrease the reversal time of a gantacurium-induced neuromuscular block at 10% recovery of T1 to a train-of-four ratio ≥ 0.90 to 3.8 min. Spontaneous reversal of the same neuromuscular block occurred in 5.7 min. Neostigmine as result of its peak effect at 7–11 min is not suitable for reversal of a gantacurium-induced neuromuscular block [12].

Gantacurium is, due to its unique metabolism, rapidly inactivated by cysteine adduction and alkaline hydrolysis. One of these routes of inactivation is adduction of the amino acid cysteine to the molecule of gantacurium. Cysteine exists as l- and d-enantiomers and the l-enantiomer is suitable as a reversal drug for the new class of neuromuscular blocking drugs: the asymmetric mixed-onium chlorofumarates [12–14, 15••]. Administration of intravenous l-cysteine results in replacement of chlorine by cysteine whereafter a heterocyclic ring is formed which cannot longer interact with the postjunctional acetylcholine receptor and the neuromuscular block can be reversed. l-Cysteine (-hydrochloric) is acidic and commonly administered in humans as an essential component of parental nutrition. l-Cysteine given in as a bolus dose of 10–50 mg/kg for reversal of neuromuscular block is not known to have any toxicity [12–14, 15••]. Therefore, gantacurium can also be reversed by administration of l-cysteine. In preclinical studies, l-cysteine administration after 8× ED_95_ of gantacurium resulted in a decrease in recovery times (to a train-of-four ratio ≥ 0.90) by 2 min. When l-cysteine was administered just 1 min after 8× ED95 of gantacurium, the recovery to a train-of-four ratio ≥ 0.90 was shortened by 6 min [12]. No signs of residual neuromuscular blockade or recurarization were observed [12]. To date, no clinical studies were conducted in which l-cysteine reversal was investigated.

### CW002

CW002 is a new benzoquinolinium fumarate diester non-depolarizing neuromuscular blocking drug of an intermediate duration of action which belongs to the family of tetrahydroisoquinolinium drugs [16, 17]. The molecular structure of CW002 is similar to that of gantacurium; the only difference is that CW002 is lacking a chlorine at the fumarate double bond and being symmetrical [16, 17]. CW002 was designed to interact more slowly with endogenous l-cysteine than gantacurium and is degraded by l-cysteine adduction and alkaline hydrolysis (t1/2 = 11.4 min). This degradation results in molecular fragments (NB 1043-10) which have 0.01–0.001 times the neuromuscular potency of CW002. l-Cysteine adduction is pH dependent [16, 17]. Furthermore, CW002 can be rapidly reversed by l-cysteine which has been shown in different animal models. Preclinical and human studies showed that a single bolus of CW002 resulted in minimal cardiopulmonary side effects and no histamine release [16, 17].

CW002 has been studied in a variety of animal models and in human volunteers and has shown initial promising results.

#### Preclinical Studies

Preclinical studies with CW002 demonstrated 100% neuromuscular blockade within 1 min and a duration of action of 27 ± 7 min in Rhesus monkeys (3.75× ED_95_) and 47 ± 9 min in dogs. This results in a ratio of 2:3 duration of action compared with cisatracurium [16, 17]. The onset times of CW002 were found to be shorter than that of cisatracurium, but longer than that of rocuronium. CW002 is inactivated by cysteine adduction and hydrolysis. The metabolite of CW002 (NB 1043-10) was 70 times less potent than CW002. In animal studies, it was shown that the ED_95_ was 0.01–0.04 mg/kg. Spontaneous recovery to a train-of-four ratio ≥ 95% occurred in about 30 min, and therefore CW002 showed an intermediate duration of action. The potency of CW002 in Rhesus monkeys differs from rocuronium as the latter showed 40% of the potency of CW002 [16, 17]. The safety of CW002 was investigated in animal models in large doses up to 10× ED_95_ and showed no significant hemodynamic changes. Only very high doses of CW002 (27 and 54× ED_95_) resulted in a 20% decrease in mean arterial pressure and a 20% increase in heart rate. Furthermore, CW002 showed low potential for bronchoconstrictive activity or histamine release [16, 17]. In Rhesus monkeys, flushing and histamine release were observed at doses up to 40 to 60× ED_95_. In contrast, animal studies with gantacurium showed histaminoid responses after doses up to 12.5 to 25× ED_95_, and histaminoid responses in humans for mivacurium and gantacurium were seen after doses of 2.5× ED_95_ and 3 to 4× ED_95_ respectively [16, 17]. Based on these preclinical results, CW002 was further investigated in humans.

#### Human Studies

The first human study investigated the dose-response and cardiopulmonary side effects of C002 [17]. The results of this study showed that CW002 is less potent in humans compared with the results seen in animal studies. The ED_95_ in humans is 0.077 mg/kg. In a dose of 1.8× ED_95_ (0.14 mg/kg) of CW002, the onset time for neuromuscular block was approximately 90 s. The clinical duration was almost 33.8 min (range 28.8–36.1 min), time from 25 to 75% recovery of T1 (interval 25–75%) 14 min. The spontaneous recovery to a train-of-four ≥ 0.90 was 73 min [17].

This study confirmed the findings shown in preclinical studies: CW002 in doses up to 0.14 mg/kg did not result in significant cardiopulmonary side effects [16–18]. Furthermore, there were no signs of histamine release [18]. As this is the first and only study in which CW002 was investigated in humans, more clinical investigations must be conducted to determine the role of CW002 in modern neuromuscular management. To date, CW002 is not available for the use in clinical practice.

#### Reversal of CW002-Induced Neuromuscular Blockade

CW002 is a non-depolarizing neuromuscular blocking drug, and therefore neuromuscular block induced by CW002 can be reversed with cholinesterase inhibitors [16–18]. Doses of neostigmine 50 μg/kg given at spontaneous recovery of T1 of 2% shortened the duration of a CW002-induced neuromuscular block minimally (from 25 to 22.5 min). Furthermore, CW002 reversal with neostigmine was significant faster than cisatracurium reversal by neostigmine (*P* < 0.05) [16–18].

CW002, like gantacurium, is inactivated rapidly by adduction of cysteine and by hydrolysis. It is to be expected that in contrast with neostigmine (in which only moderate-to-light level as of blockade can be antagonized), reversal from every level of CW002-induced neuromuscular block can be facilitated. However, this process is slower than that observed with gantacurium as duration of action of CW002 is intermediate. In vitro studies showed that the rate of l-cysteine adduction was inversely related to the duration of neuromuscular block in monkeys. Administration of l-cysteine 50 mg/kg resulted in a reversal of a CW002-induced neuromuscular block within 2–3 min when administered 60 s after 4–5× ED_95_ [16–18]. Comparative reversal of a CW002-induced neuromuscular block with cholinesterase inhibitors was significant slower. However, no study on the reversal with l-cysteine in humans is available, and therefore the clinical benefits of l-cysteine reversal of a CW002-induced neuromuscular block in humans have yet to be confirmed.

### CW011

CW011 (an asymmetrical maleate) is, as CW002 (a symmetrical fumarate), a non-halogenated olefinic diester analogue of gantacurium [12]. An in vitro study showed that this new drug had predictable slower l-cysteine adduction, and therefore a longer lasting neuromuscular blocking effect than gantacurium is to be expected [12]. In an animal study, the duration of neuromuscular block at 4–5× ED_95_ was approximately 20.8 min, half of cisatracurium duration and three times longer than that of gantacurium. CW011 showed a high potency and the calculated ED_95_ was 0.025 mg/kg. Based on this study, it was concluded that the rate of l-cysteine adduction was inversely related to the duration of action of a CW011-induced neuromuscular block in monkeys [12]. To date, no studies in humans are available and therefore the abovementioned results have to be investigated and confirmed in humans.

#### Reversal of CW011-Induced Neuromuscular Blockade

As CW011 is inactivated by cysteine adduction, CW011-induced neuromuscular block can be reversed by administration of exogenous l-cysteine. l-Cysteine (50 mg/kg) administration resulted in a significant acceleration of the recovery of a neuromuscular block induced by 4–64× ED_95_ CW011 and showed a reduction of the total duration and the 5–95% recovery interval [12]. l-Cysteine reversal showed full reversal from a neuromuscular block induced by CW011 5× ED_95_ within 2–3 min. [12] l-Cysteine reversal was superior compared with cholinesterase inhibitors in terms of speed and effectiveness of reversal of a CW011-induced neuromuscular block [12].

## Recent Advancements in Reversal of Neuromuscular Block

### Calabadion

Nowadays, pharmacological reversal of neuromuscular block can be achieved by either administering cholinesterase inhibitors or administering sugammadex. The latter encapsulates the rocuronium or vecuronium molecule and results in a rapid and effective reversal of a rocuronium- or vecuronium-induced neuromuscular block by lowering the concentration of the neuromuscular drug molecule at the neuromuscular junction. However, sugammadex is not able to reverse neuromuscular block induced by benzylisoquinolinium neuromuscular blocking drugs. In vitro and in vivo studies with calabadion 1 caused a fast and dose-dependent reversal of a cisatracurium- and rocuronium-induced neuromuscular block [20]. Calabadion 1, used in rats, in a dose of 90 mg/kg was able to reverse a rocuronium- and cisatracurium-induced neuromuscular block to recovery to a train-of-four ratio > 0.90 within 1–2 min [19, 20, 21•, 22]. There were no signs of recurarization and there were no effects on heart rate, blood pressure, or pH. Furthermore, calabadion 1 was fast eliminated by the kidney [19, 20, 21•, 22]. Calabadion 1 is also able to form an inclusion complex with local anesthetics in aqueous solution in vitro, which may play a role in the treatment of local anesthetic toxicity. However, further research is necessary to confirm this in vivo.

#### Calabadion 2

Calabadion 2 is the second-generation acyclic member of the calabadion family and chemically slightly different from calabadion 1 [20, 21•, 22]. In vitro and in vivo studies with calabadion 2 showed a higher binding affinity to rocuronium (Ka 3.4 × 10^9^ M^−1^) compared with blocking drugs [15••]. Calabadion is a recent developed drug which is able to encapsulate both steroidal and benzylisoquinolinium neuromuscular blocking drugs [15••, 19, 20, 21•, 22]. Calabadion complexes are enhancing solubility, binding affinity toward cations as a result of electrostatic interactions, and lowering the concentration of neuromuscular blocking drugs that are molecular containers and acyclic members of the cucurbituril family [19, 20, 21•, 22]. These molecules are flexible and can expand their cavity thereby able to encapsulate larger molecules like benzylisoquinolinium molecules [15••, 19, 20, 21•, 22]. Calabadions can form host-guest including rocuronium, vecuronium, and cisatracurium [15••, 19, 20, 21•, 22].

#### Calabadion 1

Calabadion 1 is the first-generation calabadion and is able to reverse neuromuscular block with the binding affinity of sugammadex to rocuronium (Ka 3.8 × 10^7^ M^−1^). Therefore, calabadion 2 showed a 89-fold stronger affinity toward rocuronium than sugammadex [20, 21•, 22]. In vivo experiments with calabadion 2 showed a faster recovery of neuromuscular blockade than reversal with calabadion 1 and more important with lower doses. Future studies are needed to confirm these findings in humans and to assess the potential side effects of calabadions in humans.

## Conclusion

In the last 20 years, research and drug development in the area of neuromuscular management were focused on a short-acting non-depolarizing neuromuscular blocking drug with a fast onset, short duration, independent of end-organ metabolism, allowing rapid and complete reversal and while safety and absence of side effects were important issues. Some interesting compounds were developed, and this novel drug development resulted in pharmacological advancements in neuromuscular management which led to a new series of compounds, chlorofumarates as new neuromuscular blocking drugs. These new compounds include gantacurium, C002, and C011 and showed promising results in onset, duration, and the potential of rapid reversal of neuromuscular blockade with l-cysteine. New advancements in reversal of neuromuscular blockade with calabadions showed interesting results in reversal of neuromuscular block induced by both steroidal and non-steroidal neuromuscular blockade. Future investigations will determine the role of these new developments in clinical practice.
